# COVID-19 fear, post-traumatic stress, growth, and the role of resilience

**DOI:** 10.1515/med-2022-0458

**Published:** 2022-04-01

**Authors:** Edita Fino, Denis Mema, Valbona Treska

**Affiliations:** Department of Experimental, Diagnostic and Specialty Medicine (DIMES), Alma Mater Studiorum-Bologna University School of Medicine, S.Orsola's University Hospital, Pad. 21 via Massarenti 9, 40138 Bologna, Italy; German Corporation for International Cooperation GmbH (GIZ), Tirana, Albania; The Order of Psychologists of Albania (OPA), Tirana, Albania

**Keywords:** COVID-19 fear, post-traumatic growth, posttraumatic stress, trait resilience

## Abstract

Understanding the factors through which pandemic fear may be associated not just with distress, but also with growth outcomes is crucial to informing interventions across population groups and cultural settings. To achieve this aim, in a cross-sectional study, we examined the relationship between the fear of COVID-19, post-traumatic stress and post-traumatic growth while assessing the moderating role of trait resilience. Findings showed that fear of COVID-19 was associated with both stress and growth outcomes and that resilience was a significant moderator of these effects. Specifically, trait resilience acted as a buffer against post-traumatic stress and as a booster factor for appreciation for life. Given the imbalance between needs and resources in times of global pandemic, interventions promoting psychological wellbeing should leverage existing resources and consider psychological resilience as a valuable target to protect against negative and optimise positive outcomes.

## 1 Introduction

Pandemics are known to elicit high levels of fear and distress [1–3] but positive reactions might also be involved as individuals strive to make sense of and adjust to adversity [4,7]. Retrospective analysis of three landmark narratives of great plagues from different historical eras has revealed common patterns in people’s responses, which included worry and distress but also a higher appreciation of relationships with others, social cohesion, and personal strength [8]. Though fear and anxiety are hallmark reactions to global pandemics, the centrality of the interpersonal and social dimension implicated in pandemic fear may cause individuals’ concerns to gravitate on affiliative and caring relationships with others [9], giving rise to a plethora of socially attuned behaviours, also known as the tend-and-befriend response to threat [10]. Caregiving relationships and social affiliation may importantly calibrate the stress response and buffer against negative effects of stress-system activation in a global pandemic context [10]. In addition, individual traits such as resilience, or one’s ability to flexibly adapt to hardship situations are believed to play a role in modulating reactions to pandemic fear and act as a protective factor for psychological wellbeing [4–7,10]. Unfortunately, most of the COVID-19 research has one-sidedly focused on the negative outcomes, leaving unexplored the examination of positive outcomes that may be associated with pandemic fear. We address this issue by examining the relationship between fear of COVID-19 with both post-traumatic stress and growth outcomes while assessing the role of trait resilience in moderating this relationship. We expected fear of pandemic to be strongly associated not only with stress but also with growth outcomes and predicted that highly resilient individuals would report lower levels of stress and higher levels of growth compared to the low resilient ones.

## 2 Method

### 2.1 Participants

A cross-sectional study was conducted from 16 to 30 December 2020 in the general population of Albania during the lockdown. Participants (18–65 years old) were invited to complete an online questionnaire investigating risk and protective factors for mental health outcomes during the lockdown. Inclusion criteria were being an adult and lacking any major physical and/or psychiatric condition. A snowball technique was employed to potentiate outreach amongst the general population. All measures used in the study were translated and back-translated from English into Albanian by accredited translators following gold standard translation practices and potential discrepancies were rectified jointly by the research team and independent bilingual individuals with work experience in psychological wellbeing issues. The study protocol and procedure were approved by the local ethics committee and all participants provided informed consent.

### 2.2 Measures

Fear of COVID-19 pandemic was measured through an adaptation of the SARS Fear Scale, [11] examining areas related to (a) the perceived risk of infection; (b) concerns about lack of healthcare due to overwhelmed facilities; and (c) concerns about restriction measures and potential consequences. In addition, items measuring concerns about psychosocial consequences of prolonged lockdown were added (sample items included “worries about the duration of quarantine”; “worries about the future” and “fear about mine/my family members’ livelihood if quarantine is prolonged”). An English version of the scale can be found here [9]. Respondents were asked to indicate the extent to which they experienced the target concerns during the last month with response options ranging from 1 (*not at all*) to 5 (*very much/extremely*). Responses were summed into a single score, with higher scores indicating higher levels of pandemic fear. The scale demonstrated a high internal consistency with Cronbach’s *α* = 0.95.

Post-traumatic stress was assessed with the post-traumatic stress disorder (PTSD) checklist for DSM-5 (PCL-5) [12] which was a 20 items scale tapping on the symptoms of post-traumatic stress (feeling very upset when something reminded you of the stressful experience and avoiding thinking about or talking about the stressful experience or avoiding having feelings related to it). Responses were rated on a 0 (*not at all*) to 4 (*extremely*) scale and were summed to provide a total severity score in the range 0–80 of PTSD symptoms. The scale demonstrated high internal consistency with Cronbach’s *α* = 0.92.

Post-traumatic growth was measured with the Post-traumatic Growth Inventory (PTGI) [13] which includes 21 items that loaded on five factors: New Possibilities (I established a new path for my life); Relating to Others (I have a greater sense of closeness with others); Personal Strength (I discovered that I’m stronger than I thought I was); Spiritual Change (I have a better understanding of spiritual matters), and Appreciation of Life (I can better appreciate each day). Respondents are asked to indicate for each item the degree of change occurring in their life as a result of the pandemic from 0 (*I did not experience this change as a result of the pandemic*) to 5 (*I experienced this change to a very great degree as a result of the crisis*), with higher scores indicating higher levels of growth. The scale showed good internal consistency with Cronbach’s *α* of 0.87 for Relating to Others, 0.85 for New Possibilities, 0.83 for Personal Strength, 0.63 for Spiritual Change, and 0.77 for Appreciation of Life.

To measure trait resilience, we used the Brief Resilience Scale (BRS) [14], which is a 6-item self-report scale with responses ranging from 1 (*strongly disagree*) to 5 (*strongly agree*). Item samples include (I tend to bounce back quickly after hard times; I have a hard time making it through stressful events). Factorial analyses assessing the dimensionality of the scale revealed a two-factor structure, with items divided according to positive/negative wording (Cronbach’s *α* of 0.60 and 0.75, respectively). The three negatively framed items were reverse-scored and summed to the rest to yield a total score with higher levels indicating a higher degree of resilience.

### 2.3 Statistical analysis

Descriptive statistics were computed to characterise the sample. To identify potential covariates to be included in subsequent analyses, differences between gender and age groups were assessed using analysis of variance. To investigate the relationship between COVID-19 fear with both stress and growth outcomes under the influence of trait resilience, we performed moderation analysis with PROCESS macro for SPSS (Model 1, with 10,000 bootstrap sampling and bias-corrected confidence intervals). The statistical significance level was set at *p* < 0.05. Analysis performed with G-Power software to determine the minimum total sample size for a medium-size effect (*f* ^2^ = 0.015) for the moderation analysis (with *p* = 0.05 and actual power = 105 0.95) was 119 participants.

## 3 Results

A total of 230 respondents completed the questionnaire. Participant characteristics for the entire sample and separately for males and females are provided in Table 1. Compared to male, female respondents reported higher levels of fear, higher levels of PTSD symptoms, and posttraumatic growth in all five dimensions.

**Table 1:** Sociodemographic and clinical characteristics of the sample

	Mean (SD); no (%)				
	Total sample (*n* = 230)	Males (*n* = 62)	Females (*n* = 189)	*F*	*p*
Age	39.9 (11.3)	39.5 (10.8)	40.1 (11.5)	0.101	0.741
Civil status				2.283	0.684
Single	105 (45.5)	26 (41.9)	78 (46.7)		
In a relationship	22 (9.5)	4 (6.4)	18 (10.6)		
Married	95 (41.1)	30 (48.3)	65 (38.4)		
Divorced/widowed	9 (3.9)	2 (3.2)	7 (4.1)		
Education				13.393	0.004
High school	21 (9.7)	12 (19.3)	9 (5.3)		
University	113 (48.9)	32 (51.6)	81 (47.9)		
Post graduate	97 (42.0)	18 (29.0)	78 (46.7)		
Fear of COVID-19	60.1 (13.1)	55.4 (12.5)	61.8 (13.0)	11.022	0.001
Post-traumatic stress (PCL_5)	19.0 (13.5)	14.8 (9.9)	20.6 (14.4)	7.921	0.005
Relationships with others (PTGI)	18.8 (7.8)	16.5 (8.3)	19.6 (7.5)	7.145	0.008
New possibilities (PTGI)	12.2 (5.8)	10.3 (5.6)	12.9 (5.8)	8.733	0.003
Personal strength (PTGI)	11.6 (4.9)	10.1 (5.0)	12.1 (4.7)	6.968	0.009
Spirituality (PTGI)	5.2 (2.8)	4.2 (2.5)	5.5 (2.8)	10.023	0.002
Appreciation for life (PTGI)	8.1 (3.4)	6.6 (3.5)	8.6 (3.2)	16.892	<0.001
Trait Resilience (BRS)	3.5 (0.6)	3.5 (0.6)	3.5 (0.6)	0.283	0.595

Note: PCL-5: Post-traumatic stress Checklist for DSM-5; PTGI: Post-traumatic Growth Index; BRS: Brief Resilience Scale.

**Figure 1:**
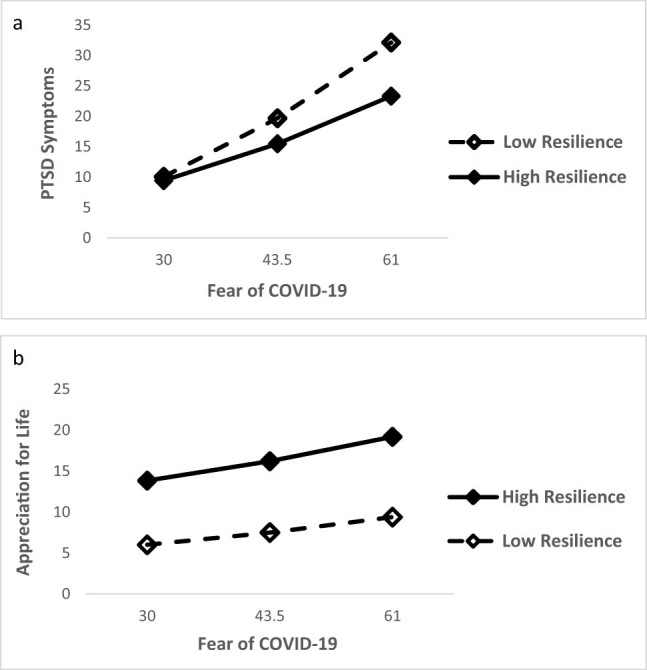
Trait resilience moderates the relationship between COVID-19 fear with both (a) post-traumatic stress and (b) and post-traumatic growth.

Moderation analyses were separately performed with post-traumatic stress and post-traumatic growth set as the outcome (Y) respectively, COVID-19-related fear set as the predictor (X) and trait resilience set as moderator (M), controlling for gender and age. The full model was significant for PTSD: *R* ^2^ = 0.48, MSE = 95.936, *F*_3,227_ = 66.385, *p* < 0.0001 and the interaction between COVID-19 fear and resilience was significant: *β* = −0.231, SE = 0.0661, *t* = −3.463, *p* < 0.001; 95% CI [−0.362, −0.099]. As indicated by respective slopes in Figure 1a, the higher the level of trait resilience, the lower the level of PTSD symptoms. Notably, as fear of COVID-19 increases, the protective role of trait resilience becomes crucial in discriminating those more susceptible to developing clinically relevant symptoms of PTSD (cut off ≥ 31) from those that don’t.

The same analysis performed with post-traumatic growth subscales set as outcome variables yielded a significant full model for Appreciation for Life: *R* ^2^ = 0.21, MSE = 9.445, *F*_3,227_ = 19.049, *p* < 0.0001. The trait resilience by fear of COVID-19 interaction was significant: *β* = −0.045, SE = 0.021, *t* = −2.132, *p* = 0.03; 95% CI [−0.086, −0.003]. Respective slopes shown in Figure 1b indicate that trait resilience has a significant impact on post-traumatic growth across levels of pandemic fear – that is, higher the resilience, stronger the appreciation for life.

## 4 Discussion

There is a heightened interest in understanding the factors through which pandemic fear may lead not just to negative but also positive outcomes across population groups and cultural settings to better inform community-based interventions [15]. In this study, we found that fear of COVID-19 was associated with both stress and growth outcomes and this relationship was moderated by trait resilience. Specifically, highly resilient individuals reported lower levels of PTSD and higher levels of appreciation for life during the lockdown. These findings advance current knowledge on the role of resilience as a protective factor for psychological wellbeing in the context of adversity [11] and global pandemics [4–7], by examining pathways from pandemic fear to both stress and growth outcomes in the general population. However, some limitations should be mentioned. For instance, the convenience sample and cross-sectional nature of the study might limit the generalizability of the results. The fact that our sample was composted by mostly female participants, who were on average rather young and well-educated may have further influenced our results. Notwithstanding these limitations, this study importantly suggests that in a pandemic, fear and distress can be high but there is also a potential for growth.

While the role of pandemic fear as a risk factor for distress should not be overlooked, the role of protective factors in harnessing the potential for growth in the face of adversity should also be considered. Considering the imbalance between needs and resources in times of global pandemic, community-based interventions promoting psychological wellbeing should leverage existing resources and consider psychological resilience as a valuable target to protect against negative and optimise positive outcomes.

## References

[1] Boyraz G, Legros DN, Tigershtrom A. COVID-19 and traumatic stress: the role of perceived vulnerability, COVID-19-related worries, and social isolation. J Anxiety Disord. 2020;76:102307. 10.1016/j.janxdis. PMC7831572 32937259

[2] Fitzpatrick KM, Harris C, Drawve G. Fear of COVID-19 and the Mental Health Consequences in America. Psychol Trauma. 2020;12(S1):S17–21. 10.1037/tra0000924 32496100

[3] Pérez-Fuentes M, Jurado MD, Martínez ÁM, Linares JJ. Threat of COVID-19 and emotional state during quarantine: positive and negative affect as mediators in a cross-sectional study of the Spanish population. PLoS One. 2020;15(6):1–11. 10.1371/journal.pone.0235305 PMC7316299 32584897

[4] Fino E, Bonfrate I, Fino V, Bocus P, Russo PM, Mazzetti M. Harnessing distress to boost growth in frontline healthcare workers during COVID-19 pandemic: the protective role of resilience, emotion regulation and social support. Psychol Med. 2021;1–9. 10.1017/S0033291721000519. PMC7900668 33565390

[5] Bonanno GA, Ho SM, Chan JC, Kwong RS, Cheung CK, Wong CP, et al. Psychological resilience and dysfunction among hospitalized survivors of the SARS epidemic in Hong Kong: a latent class approach. Health Psychol. 2008;27(5):659–67. 10.1037/0278-6133.27.5.659 18823193

[6] Killgore W, Taylor EC, Cloonan SA, Dailey NS. Psychological resilience during the COVID-19 lockdown. Psychiatry Res. 2020;291:113216. 10.1016/j.psychres.2020.113216. PMC7280133 32544705

[7] Paredes MR, Apaolaza V, Fernandez-Robin C, Hartmann P, Yañez-Martinez D. The impact of the COVID-19 pandemic on subjective mental well-being: The interplay of perceived threat, future anxiety and resilience. Pers Individ Dif. 2021;170:110455. 10.1016/j.paid.2020.110455. PMC7552984 33071413

[8] Wigand ME, Becker Th, Steger F. Psychosocial reactions to plagues in the cultural history of medicine a medical humanities approach. J Nerv Ment Dis. 2020;208:443–4. 10.1097/NMD.0000000000001200 32472811

[9] Fino E, Mema D, Treska V. The interpersonal dimension of pandemic fear and the dual factor model of mental health: The role of coping strategies. Healthcare. 2022;10(2):247. 10.3390/healthcare10020247. PMC8871641 35206862

[10] Taylor SE. Affiliation and stress. In: Folkman S, editor. Oxford handbook of stress, health, and coping. New York, NY: Oxford University Press; 2012. p. 86–100.

[11] Ho SMY, Kwong-Lo RSY, Mak CWY, Wong JS. Fear of severe acute respiratory syndrome (SARS) among health care workers. J Consult Clin Psychol. 2005;73(2):344–9. 10.1037/0022-006X.73.2.344 15796643

[12] Weathers FW, Litz BT, Keane TM, Palmieri PA, Marx BP, Schnurr PP. The PTSD Checklist for DSM-5 (PCL-5).2013. Scale available from the National Center for PTSD at www.ptsd.va.gov.

[13] Tedeschi RG, Calhoun LG. The posttraumatic growth inventory: measuring the positive legacy of trauma. J Trauma Stress. 1996;9:455–71. 10.1007/BF02103658 8827649

[14] Smith BW, Dalen J, Wiggins K, Tooley E, Christopher P, Bernard J. The brief resilience scale: assessing the ability to bounce back. Int J Behav Med. 2008;15(3):194–200. 10.1080/10705500802222972 18696313

[15] Holmes EA, O’Connor RC, Perry VH, Tracey I, Wessely S, Arseneault L, et al. Multidisciplinary research priorities for the COVID-19 pandemic: a call for action for mental health science. Lancet Psychiat. 2020;7(6):547–60. 10.1016/S2215-0366(20)30168-1 PMC7159850 32304649

